# Applied forces during neonatal intubation with direct and video laryngoscopy at different bed elevations: a randomized crossover manikin study

**DOI:** 10.1007/s00431-025-06524-8

**Published:** 2025-11-05

**Authors:** Francesco Cavallin, Greta Pasquali, Sabina Maglio, Paolo Ernesto Villani, Arianna Menciassi, Selene Tognarelli, Daniele Trevisanuto

**Affiliations:** 1 Solagna, Italy; 2 Department of Woman’s and Child’s Health, University Hospital of Padua, Via Giustiniani, 3, Padua, 35128 Italy; 3 The BioRobotics Institute, Scuola Superiore Sant’Anna, Pisa, Italy; 4 Department of Excellence in Robotics & AI, Scuola Superiore Sant’Anna, Pisa, Italy; 5 Department of Woman’s and Child’s Health, Poliambulanza Hospital, Fondazione Poliambulanza, Brescia, Italy; 6 Department of Women and Children Health, University of Padova, Padua, Italy

**Keywords:** Force, Intubation, Manikin, Neonate

## Abstract

We compared intubation forces, success of the first attempt, intubation time, and participant’s opinions using direct and video laryngoscopy at umbilical and xiphoidal bed heights in a neonatal manikin. A randomized controlled crossover trial of intubation using direct and video laryngoscopes at two different table elevations (operator’s xiphoid or navel) in a neonatal manikin model. Thirty-two neonatologists and pediatric residents participated. The primary outcome measure was the force applied to epiglottis and palate of neonatal manikin during intubation. The secondary outcome measures included success of the first attempt, intubation time, and participants’ opinion on the procedures. Intubating the neonatal manikin with the videolaryngoscope and the resuscitation table leveled at operator’s navel was associated with the lowest forces applied to the epiglottis and the hard palate. On the other hand, the combination of direct laryngoscope and resuscitation table leveled at operator’s xiphoid was associated with the highest applied forces. The success rate at the first attempt and the intubation time were not statistically different among the combinations of laryngoscopes and table elevations. In contrast to actual measurements, most participants believed to have used less forces when the table leveled at operator’s xiphoid.

*Conclusions*: In a neonatal manikin model, the lower force applied during intubation with a videolaryngoscope and the resuscitation table leveled at operator’s navel may be a desirable objective, but the clinical implications should be evaluated in clinical studies.

*Trial registration*:clinicaltrial.gov NCT06474572

**Table:** 

**What is Known:** • *Intubation success may be influenced by procedure-related aspects such as the position of the patient in relation with the operator*. • *The Neonatal Resuscitation Program recommends adjusting the bed height to align the baby's head with operator's upper abdomen or lower chest, while positioning the bed height at approximately elbow height is also suggested*. • *The bed height might affect the applied forces during neonatal intubation and the intubation success, but such information is currently lacking*.
**What is New:** • *In a neonatal manikin model, intubating with a videolaryngoscope and the resuscitation table leveled at operator’s navel was associated with the lowest forces applied to the epiglottis and the hard palate*. • *The participants achieved a high success rate at the first attempt and comparable procedure times with all combinations of direct/video laryngoscope and low/high bed elevation*. • *Applying lower forces during neonatal intubation may be desirable but the clinical implications remain to be evaluated in clinical studies*.

**Supplementary Information:**

The online version contains supplementary material available at 10.1007/s00431-025-06524-8.

## Introduction

Intubating at birth is required in approximately 1% of neonates [1]. Nonetheless, health care providers have limited exposure to neonatal intubation because of the implementation of less invasive procedures, such as continuous positive airway pressure and nasal intermittent positive pressure ventilation [2, 3].

Laryngoscopy is an invasive and potentially harmful procedure, which has been associated with adverse events including airway trauma, bradycardia, hypoxia, and intraventricular hemorrhage [4–6]. Videolaryngoscopy improves the intubation success at the first attempt compared to direct laryngoscopy in neonates and seems to decrease the incidence of adverse events [7–9].

During the intubation procedure, the movements create compression forces on the soft tissues that can cause tissue ischemia or perforation, adverse clinical reactions, or future developmental issues in the surrounding structures [4, 6, 7, 9–11]. According to a neonatal manikin trial, lower forces are applied with videolaryngoscopy compared to direct laryngoscopy, suggesting possible benefits in reducing patient harm during intubation [12].

Intubation success may be influenced by procedure-related aspects such as the position of the patient in relation with the operator. In adults, the literature offers conflicting findings, which include suggesting the bed height to the level of operator’s xiphoid [13–15], navel [14, 16], or no preference among different bed height [17, 18]. The Neonatal Resuscitation Program recommends adjusting the bed height to align the baby’s head with operator’s upper abdomen or lower chest [11], while a recent study suggested positioning the bed height at approximately elbow height [19]. We assumed that the bed height might affect the applied forces during neonatal intubation, but such information is currently lacking.

This trial compared intubation forces applied with direct and video laryngoscopy at umbilical and xiphoidal bed heights in a neonatal manikin. Furthermore, success of the first attempt, intubation time and participant’s opinions about the procedures were investigated.

## Methods

### Study design

This was a randomized, controlled, crossover trial of intubation using direct and video laryngoscopes at two different table elevations in a neonatal manikin model (clinicaltrials.gov NCT06474572). The trial employed a 4-sequence, 4-period, 4-treatment scheme (ADBC/BACD/CBDA/DCAB) which is uniform within sequences and periods, and balanced with respect to first-order carryover effects [20]. The Ethics Committees of the University Hospital of Padua (Italy) reviewed and approved this manikin study (Prot. 536n/AO/24). The participants provided their written informed consent.

### Setting

**Fig. 1:**
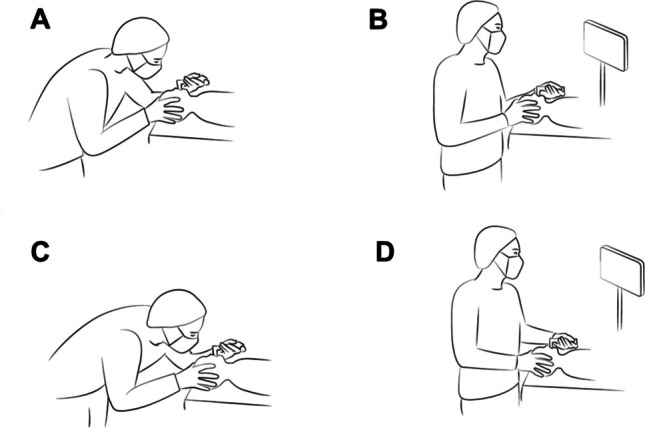
Participants performed the intubation with a direct laryngoscope and the resuscitation table height adjusted to level operator’s xiphoid (**A**), a videolaryngoscope and the resuscitation table height adjusted to level operator’s xiphoid (**B**), a direct laryngoscope and the resuscitation table height adjusted to level operator’s navel (**C**), and a videolaryngoscope and the resuscitation table height adjusted to level operator’s navel (**D**)

This simulation study was performed at the University Hospital of Padua (Italy) between 1 st and 5th July 2024. In the simulation, participants were asked to intubate a full-term neonatal manikin (NewBorn Anne, Laerdal, Stavanger, Norway) using a standard direct laryngoscope with Miller blade size 1 and a videolaryngoscope with Miller blade Spectrum S1 (Verathon Inc., Bothell, WA, USA) at two different table elevations to level operator’s xiphoid or navel (Fig. 1). This manikin is a task trainer specifically developed for the acquisition of cardiopulmonary resuscitation skills, including endotracheal intubation.

### Participants

Eligible participants were level III neonatal intensive care unit (NICU) consultants and pediatric residents. Refusal to participate was the only exclusion criteriom.

### Randomization

All participants were randomly assigned to one of the four sequences in a 1:1:1:1 ratio. Randomization was performed using a computer-generated random assignment list. Arm assignments were put in sequentially numbered, sealed, opaque envelopes.

### Procedures

Participants in ADBC arm were assigned to perform the intubation with a direct laryngoscope and the resuscitation table height adjusted to level operator’s xiphoid (A), followed by the intubation with a videolaryngoscope and the resuscitation table height adjusted to level operator’s navel (D), by the intubation with a videolaryngoscope and the resuscitation table height adjusted to level operator’s xiphoid (B), and by the intubation with a direct laryngoscope and the resuscitation table height adjusted to level operator’s navel (C). Participants in different arms were assigned to perform the intubations in different sequences (ADBC/BACD/CBDA/DCAB). A washout period of three hours was included to reduce any carryover effect.

Before the simulation, an expert in laryngoscopy intubation showed the intubation techniques performed with a videolaryngoscope and a laryngoscope equipped with a Miller blade on a neonatal manikin. Each participant practiced with both devices on the manikin before the study sessions.

In the simulation, participants were asked to intubate the neonatal manikin with a 3.5-mm endotracheal tube using both laryngoscopes at two different table elevations to level operator’s xiphoid or navel. During each intubation attempt, a researcher documented the number of intubation attempts and the total intubation time. An intubation attempt was considered as failed if the endotracheal tube was not positioned in the trachea or if the attempt lasted more than 60 s [9].

During each procedure, force measurements were acquired using three force sensors (FlexiForce A301; Tekscan, Inc., Norwood, MA, USA) as previously described [12]. One sensor (epiglottic sensor) was placed on the distal surface of the blade in correspondence to the area in contact with the epiglottis during intubation and two sensors (palatal sensors) on the proximal surface of the blade at the area in touch with the upper gum and hard palate [12].

### Outcome measures

The primary outcome measure was the measurement of the forces (peak, average peak, and standard deviation) applied to the epiglottis and the palate of the neonatal manikin during the intubation. The forces were expressed as Newton (N). The secondary outcome measures included the success of the first attempt, the total time of intubation (calculated as the sum of the duration of all intubation attempts) and participant’s opinions about the procedures. Participants were asked (i) to indicate the preferred table elevation during the simulation, (ii) to rate the difficulty in intubating with the two table elevations using a 5-point Likert scale, (iii) to report troubles (postural discomfort, visualizing the anatomic structures, positioning the endotracheal tube, and managing the laryngoscope) with the two table elevations, (iv) to indicate the preferred laryngoscope at each table elevation, and (v) to state which table elevation was associated with less applied forces in their perception.

### Data collection and measurements

All data were collected by an observer who was not involved in the simulation. Data were recorded on a data sheet designed for the study and stored in a password-protected computer. Data collection included participant characteristics (demographics, experience in neonatal or pediatric intensive care, experience with direct laryngoscopy and video laryngoscopy, clinical routine about table elevation during intubation) and data on the outcome measures (applied forces, success of the first attempt, the total time of intubation and participant’s opinions about the procedures). Participant’s opinions were collected at the end of the simulation.

### Blinding

The participants and the outcome assessors could not be masked due to the characteristics of the interventions. However, the statistician who analyzed the data was masked to treatment allocation.

### Statistical analysis

As we were unable to predict the magnitude of the difference in forces applied with the different combinations of laryngoscope and table height, a formal sample size calculation could not be performed during study planning and a convenience sample size of 32 participants was chosen for the trial. In the analysis of the applied forces, 50th percentile (median) and top 10th percentile were calculated as relevant indicators of peak force, average force, and standard deviation of applied force, while median and top 10th percentile of the paired differences between two groups were used for comparisons. The top 10th percentile difference in forces was chosen to assess the maximum difference in forces [12, 21]. Bootstrap confidence intervals (CI) were calculated for percentiles and differences, and any CI for the difference not including zero suggested a statistically significant difference. Because of coverage error of bootstrap CIs for percentiles in small-sized samples, empirical bootstrap 99% CIs were calculated using re-sampling with replacement to create 1000 samples of the same size as the original [21]. In the analysis of the total time of intubation, the median was calculated as relevant indicator and the median of the paired differences between two groups were used for comparisons. Bootstrap CIs were calculated and interpreted as described above. The success rate at the first attempt was reported as absolute and relative frequency (percentage), and the difference in proportion for paired data was used for comparisons between two groups. Any 95% CI for the difference not including zero suggested a statistically significant difference. Participants’ opinions on the difficulty associated with the two table elevations were measured using a Likert scale and compared using the Wilcoxon test. Overall, participants’ opinions on the procedures were summarized using descriptive statistics. Statistical analysis was performed using R 4.4 (R Foundation for Statistical Computing, Vienna, Austria) [22].

## Results

### Participants

The study included 32 participants (23 females and nine males) with a median age of 32 years (IQR 30–44). They were 15 NICU consultants (47%) and 17 pediatric residents (53%). Median experience in neonatal or pediatric intensive care units was 5 years (IQR 4–10). Twenty participants (63%) were most acquainted with the direct laryngoscopy, eight (25%) with the videolaryngoscopy, and two (6%) with both, while other two (6%) did not provide such information.

Experience with direct laryngoscopy was > 10 intubations in 16 participants (50%), 1–10 intubations in nine participants (28%), and none in six participants (19%). Experience with videolaryngoscopy was > 10 intubations in six participants (19%), 1–10 intubations in 19 participants (59%), and none in six participants (19%). One participant (3%) did not provide such information.

During their clinical activity, the participants reported to check the table elevation before performing an intubation always (*n* = 16), often (*n* = 7), sometimes (*n* = 5), and rarely (*n* = 3), while one participant did not provide such information. In addition, four of them (13%) usually adjust the table to level their xiphoid and four (13%) to level their navel, while 22 (68%) declared to opt for an intermediate elevation between the xiphoid and the navel (two participants, 6%, did not provide such information).

Complete data for the outcome measures were obtained for all participants. All participants performed the allocated sequence and there was no loss to follow-up (Supplementary Figure S1).

### Applied forces

**Table 1:** Primary outcome measures: forces applied by the participants during the intubation

Area	Outcome	Percentile	Direct laryngoscope and table at xiphoidal level (A)	Videolaryngoscope and table at xiphoidal level (B)	Direct laryngoscope and table at umbilical level (C)	Videolaryngoscope and table at umbilical level (D)	Paired difference (A–D)	Paired difference (B–D)	Paired difference (C–D)
Epiglottic sensor	Peak	Median (bootstrap 99% CI)	5.6 (4.6 to 7.0)	3.5 (3.0 to 5.4)	4.4 (3.6 to 4.9)	3.1 (2.4 to 4.2)	2.3 (1.6 to 4.3)	1.2 (−0.4 to 2.1)	1.5 (0.6 to 2.1)
		Top 10th percentile (bootstrap 99% CI)	8.6 (6.9 to 15.3)	9.1 (5.3 to 10.1)	6.0 (4.8 to 7.9)	5.2 (4.1 to 7.0)	5.0 (4.2 to 10.9)	3.9 (2.0 to 8.1)	2.6 (2.0 to 4.5)
	Average peak	Median (bootstrap 99% CI)	3.4 (2.7 to 3.8)	2.7 (1.5 to 4.0)	2.5 (2.0 to 3.3)	1.7 (1.2 to 2.6)	1.7 (0.5 to 2.8)	0.9 (0.1 to 1.6)	0.7 (0.1 to 1.5)
		Top 10th percentile (bootstrap 99% CI)	6.1 (3.7 to 7.1)	5.7 (3.8 to 6.1)	4.2 (3.2 to 6.1)	3.7 (2.5 to 4.9)	3.2 (2.8 to 4.5)	3.3 (1.3 to 5.1)	2.7 (1.3 to 3.8)
	Standard deviation	Median (bootstrap 99% CI)	1.8 (1.2 to 2.1)	1.0 (0.8 to 1.6)	1.2 (1.1 to 1.5)	0.9 (0.7 to 1.2)	0.7 (0.2 to 1.2)	0.2 (−0.2 to 0.6)	0.3 (−0.1 to 0.6)
		Top 10th percentile (bootstrap 99% CI)	2.7 (2.1 to 3.2)	2.2 (1.6 to 3.3)	1.8 (1.5 to 3.0)	1.5 (1.2 to 1.9)	1.5 (1.1 to 2.0)	1.5 (0.5 to 1.8)	1.1 (0.5 t 1.6)
Palatal sensor in contact with the hard palate	Peak	Median (bootstrap 99% CI)	1.0 (0.0 to 2.8)	0 (0.0 to 3.8)	0.9 (0.0 to 3.0)	0.4 (0.0 to 3.0)	0.0 (−1.1 to 0.9)	0.0 (0.0 to 0.1)	0.0 (−1.7 to 1.6)
		Top 10th percentile (bootstrap 99% CI)	4.8 (2.8 to 7.0)	9.7 (1.8 to 14.5)	4.3 (3.0 to 6.0)	9.1 (1.9 to 13.8)	3.8 (0.6 to 6.9)	5.4 (0.0 to 9.2)	3.3 (0.4 to 3.9)
	Average peak	Median (bootstrap 99% CI)	0.7 (0.0 to 1.8)	0.0 (0.0 to 1.1)	0.7 (0.0 to 1.9)	0.3 (0.0 to 2.1)	0.0 (−0.9 to 0.9)	0.0 (−0.5 to 0.3)	0.0 (−0.3 to 0.7)
		Top 10th percentile (bootstrap 99% CI)	3.6 (1.7 to 5.4)	4.0 (0.9 to 7.5)	2.9 (1.8 to 3.9)	4.2 (1.7 to 9.1)	2.3 (0.6 to 5.3)	1.9 (0.0 to 5.5)	1.8 (0.4 to 2.6)
	Standard deviation	Median (bootstrap 99% CI)	0.2 (0.0 to 1.0)	0.0 (0.0 to 0.5)	0.3 (0.0 to 0.9)	0.1 (0.0 to 0.8)	0.0 (−0.3 to 0.4)	0.0 (−0.1 to 0.2)	0.0 (−0.4 to 0.5)
		Top 10th percentile (bootstrap 99% CI)	1.8 (0.8 to 2.4)	2.7 (0.5 to 4.0)	1.4 (0.9 to 2.3)	2.0 (0.6 to 4.2)	1.3 (0.2 to 2.3)	1.2 (0.0 to 2.6)	1.1 (0.5 to 1.3)
Palatal sensor in contact with the upper gum	Peak	Median (bootstrap 99% CI)	0.0 (0.0 to 0.0)	0.0 (0.0 to 0.0)	0.0 (0.0 to 0.0)	0.0 (0.0 to 0.0)	0.0 (0.0 to 0.0)	0.0 (0.0 to 0.0)	0.0 (0.0 to 0.0)
		Top 10th percentile (bootstrap 99% CI)	0.6 (0.0 to 1.8)	6.8 (0.0 to 11.7)	0.8 (0.0 to 8.7)	0.7 (0.0 to 13.3)	0.6 (0.0 to 1.8)	0.7 (0.0 to 11.6)	0.7 (0.0 to 8.7)
	Average peak	Median (bootstrap 99% CI)	0.0 (0.0 to 0.0)	0.0 (0.0 to 0.0)	0.0 (0.0 to 0.0)	0.0 (0.0 to 0.0)	0.0 (0.0 to 0.0)	0.0 (0.0 to 0.0)	0.0 (0.0 to 0.0)
		Top 10th percentile (bootstrap 99% CI)	0.4 (0.0 to 1.0)	2.4 (0.0 to 7.8)	0.4 (0.0 to 9.0)	0.7 (0.0 to 4.6)	0.4 (0.0 to 1.0)	0.6 (0.0 to 7.6)	0.3 (0.0 to 8.5)
	Standard deviation	Median (bootstrap 99% CI)	0.0 (0.0 to 0.0)	0.0 (0.0 to 0.0)	0.0 (0.0 to 0.0)	0.0 (0.0 to 0.0)	0.0 (0.0 to 0.0)	0.0 (0.0 to 0.0)	0.0 (0.0 to 0.0)
		Top 10th percentile (bootstrap 99% CI)	0.1 (0.0 to 0.6)	1.8 (0.0 to 4.1)	0.2 (0.0 to 2.2)	0.2 (0.0 to 2.8)	0.1 (0.0 to 0.6)	0.2 (0.0 to 4.1)	0.2 (0.0 to 2.2)

Any CI for the difference between forces not including zero indicated a statistically significant difference

 Table 1 summarizes the forces applied by the participants during the procedures.

The epiglottic sensor recorded higher median and top 10th percentile of applied forces (peak, average peak, and standard deviation) using the direct laryngoscope with the table at xiphoidal or umbilical level vs. the videolaryngoscope with the table at umbilical level. The epiglottic sensor also recorded higher median of applied forces (average peak) and higher top 10th percentile of applied forces (peak, average peak, and standard deviation) using the videolaryngoscope when the table was set at xiphoidal vs. umbilical level.

The hard palate sensor recorded higher top 10th percentile of applied forces (peak, average peak, and standard deviation) using the direct laryngoscope with the table at xiphoidal or umbilical level vs. the videolaryngoscope with the table at umbilical level.

The upper gum sensor did not record any statistically significant difference between the videolaryngoscope with the table at umbilical level and the other combinations in terms of applied forces.

All numerical results are displayed in Table 1.

#### Success rate at the first attempt

Table 2 shows the success rate at the first attempt. Using the direct laryngoscope, the success rate was 84% with the table at xiphoidal level and 91% with the table at umbilical level. Using the videolaryngoscope, the success rate was 97% with both table elevations. The success rate at the first attempt was not statistically different between the videolaryngoscope with the table at umbilical level and the other combinations (Table 2).

**Table 2:** Secondary outcome measure: intubation success at the first attempt

Outcome	Direct laryngoscope and table at xiphoidal level (A)	Videolaryngoscope and table at xiphoidal level (B)	Direct laryngoscope and table at umbilical level (C)	Videolaryngoscope and table at umbilical level (D)	Difference in proportion for paired data (95% confidence interval) (A-D)	Difference in proportion for paired data (95% confidence interval) (B-D)	Difference in proportion for paired data (95% confidence interval) (C-D)
Success at the first attempt, n (%)	27/32 (84%)	31/32 (97%)	29/32 (91%)	31/32 (97%)	−13% (−29% to 3%)	0%−14% to 14%)	−6% (−21% to 7%)

Any CI for the difference between forces not including zero indicated a statistically significant difference

#### Total intubation time

Median total intubation time ranged from 17.5 s using the videolaryngoscope with the table at umbilical level to 19.5 s using the direct laryngoscope with the table at xiphoidal level (Table 3). The total intubation time was not statistically different between the videolaryngoscope with the table at umbilical level and the other combinations (Table 3).

**Table 3:** Secondary outcome measure: total intubation time

Outcome	Percentile	Direct laryngoscope and table at xiphoidal level (A)	Videolaryngoscope and table at xiphoidal level (B)	Direct laryngoscope and table at umbilical level (C)	Videolaryngoscope and table at umbilical level (D)	Paired difference (A-D)	Paired difference (B-D)	Paired difference (C-D)
Total intubation time, seconds	Median (bootstrap 99% CI)	19.5 (15.0 to 23.0)	18.0 (14.5 to 23.0)	19.0 (14.0–23.0.0.0)	17.5 (15.0 to 22.0)	2 (−3 to 4)	0 (−2 to 3)	0.5 (−2 to 4)

Any CI for the difference between forces not including zero indicated a statistically significant difference

#### Participants’ opinions on the procedures

Overall, 17 participants (53%) indicated the xiphoidal level and 15 (47%) the umbilical level as preferred table elevation during the simulation. They scored a median difficulty of 2 out of max 5 points (IQR 2–3) with the table at xiphoidal level and 3 out of max 5 points (IQR 2–3) with the table at umbilical level (*p* = 0.94). The reported troubles with the table at xiphoidal level included postural discomfort (*n* = 12), visualizing the anatomic structures (*n* = 5), positioning the endotracheal tube (*n* = 5), and managing the laryngoscope (*n* = 3). The reported troubles with the table at umbilical level included postural discomfort (*n* = 10), visualizing the anatomic structures (*n* = 9), and positioning the endotracheal tube (*n* = 5).

When the table leveled the operator’s xiphoid, 17 participants (53%) preferred the videolaryngoscope and 15 (47%) the direct laryngoscope. When the table leveled the operator’s navel, 24 participants (75%) preferred the videolaryngoscope and 8 (25%) the direct laryngoscope.

Overall, 23 participants (72%) believed to have used less force with the table at xiphoidal level and 9 (28%) with the table at umbilical level.

**Fig. 2:**
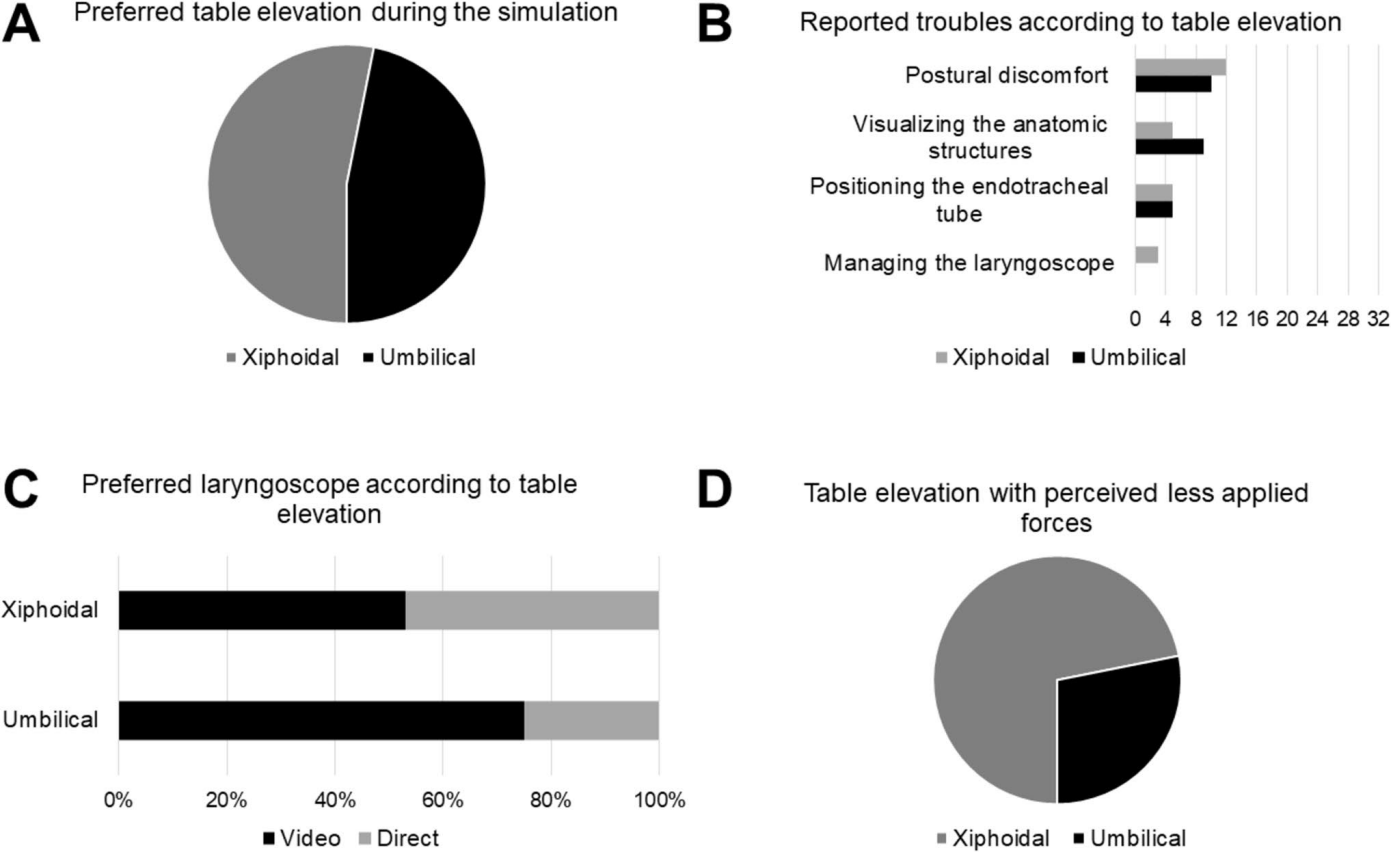
Visual summary of participants’ opinions on the procedures: the participants slightly preferred intubating with the table at xiphoidal level (**A**); they reported different troubles according to the table elevation (**B**); the videolaryngoscope was largely preferred to the direct laryngoscope with the table at umbilical level (**C**); most participants believed to have used less force with the table at xiphoidal level (**D**)

Figure 2 offers a visual summary of participants’ opinions on the procedures.

## Discussion

Our trial compared intubation with direct and video laryngoscopy in a neonatal manikin positioned at umbilical and xiphoidal bed heights. We hypothesized that the combination of direct/video laryngoscopy and bed height might influence the applied forces during neonatal intubation.

The literature suggests that videolaryngoscopy may involve lower applied forces compared to direct laryngoscopy in neonatal resuscitation, while the preferred elevation of the resuscitation table remains controversial [11, 12, 19]. Our investigation provides further information about the potential differences in the forces applied by the resuscitator according to the combination of laryngoscope and bed height. Overall, our findings suggested that intubating a neonatal manikin with the videolaryngoscope and the resuscitation table leveled at operator’s navel was associated with the lowest forces applied to the epiglottis and the hard palate. On the other hand, the combination of direct laryngoscope and resuscitation table leveled at operator’s xiphoid was associated with the highest applied forces to the epiglottis and the hard palate. These results confirmed previous observations about the lower applied forces when using videolaryngoscopy compared to direct laryngoscopy [12]. Furthermore, we hypothesize that a high table elevation may push the operator to act beyond the ergonomic levels, probably affecting the applied forces to perform the intubation maneuver. Of note, our data revealed that subjective perceptions of applied forces differed from the actual measurements, as most participants believed to have used less forces when the table leveled operator’s xiphoid. Such discrepancy may advocate for the implementation of a technology capable to provide force feedback to the operator during the intubation. Nevertheless, the reader should be aware that the magnitude of harmful applied forces during intubation remains unknown.

Videolaryngoscopy has been associated with higher intubation success at the first attempt compared to direct laryngoscopy in neonates [7–9]. In our simulation, the participants achieved a high success rate at the first attempt with all combinations of laryngoscope and bed height, alongside with comparable intubation times. Interestingly, the confidence intervals did not exclude that videolaryngoscopy might improve the success rate compared to direct laryngoscopy irrespectively of bed height.

Noteworthy, one bed height did not prevail over the other according to participants’ preferences. This finding might be influenced by their clinical routine, when the resuscitation table was mostly set at an intermediate elevation between the xiphoid and the navel. Previous studies suggested that higher table positions (such as xiphoid or nipple level) might provide better laryngeal views and less operator’s discomfort when intubating with a direct laryngoscope, while umbilical table position might warrant better visibility and less shoulder joint flexion when using a videolaryngoscope [13, 14]. Our participants confirmed to prefer videolaryngoscopy with the umbilical table position, while one device did not prevail over the other with the xiphoidal table position.

To our knowledge, this is the first trial investigating the combination of direct/video laryngoscopy and bed height during a simulated neonatal intubation.

The strengths of our trial included the crossover design, the objective and reliable force measurement, and the enrolment of participants with heterogeneous experience with laryngoscopy and resuscitation table elevation.

However, some limitations should be considered when reading the results. First, manikin tissue and anatomy could not replicate the exact texture, flexibility, and responsiveness of a living human body, and eliminated the anatomical heterogeneity among neonates [12]. Consequently, its use should be regarded primarily as a procedural simulation employing an inert task trainer, intended for the acquisition and repetition of technical skills rather than the reproduction of realistic anatomical conditions. Second, the applied forces may differ in a real-life intubation. Third, the trial included only one model of neonatal manikin and two elevations of the resuscitation table. Lastly, the generalizability of the findings should be limited to operators with comparable experience.

## Conclusions

In a neonatal manikin model, intubating with a videolaryngoscope and the resuscitation table leveled at operator’s navel was associated with the lowest forces applied to the epiglottis and the hard palate. The participants achieved a high success rate at the first attempt and comparable procedure times with all combinations of direct/video laryngoscope and low/high bed elevation. Subjective perceptions of applied forces differed from the actual measurements. Applying lower forces during neonatal intubation may be desirable but the clinical implications remain to be evaluated in clinical studies.

## Supplementary Information

ESM 1 (PNG 311 KB)

High resolution image (TIF 995 KB)

## Data Availability

All data generated or analyzed during this study are included in this article. Further inquiries can be directed to the corresponding author.

## Abbreviations

IQR: Interquartile range
N: Newton
NICU: Neonatal intensive care unit
