# Supplementary figures and images for: Applied forces during neonatal intubation with direct and video laryngoscopy at different bed elevations: a randomized crossover manikin study
Source: Eur J Pediatr. 2025 Nov 5;184(12):732. doi: 10.1007/s00431-025-06524-8 (PMC12589294; doi:10.1007/s00431-025-06524-8)

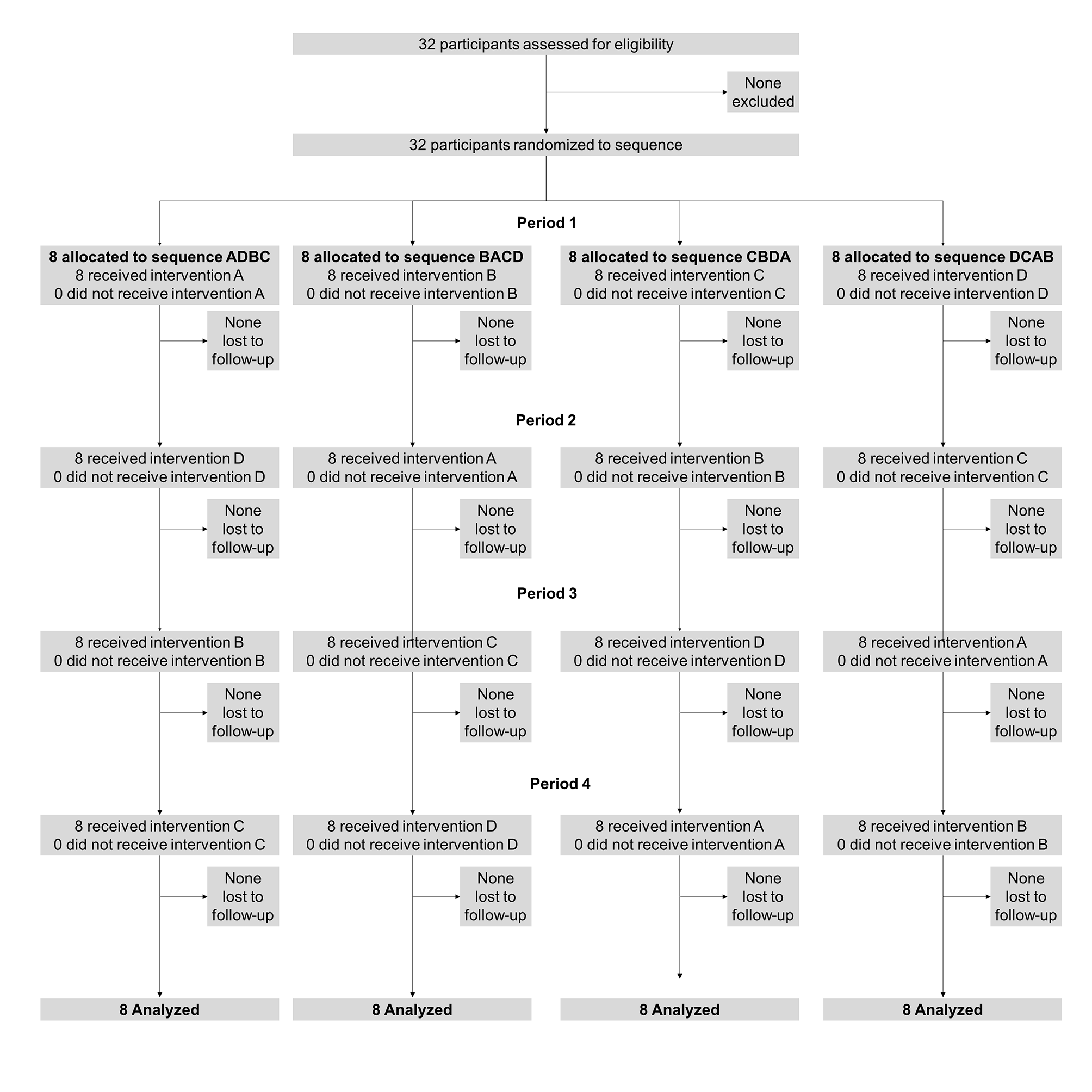

Supplement: Supplementary file 1 — (PNG 311 KB) [file 431_2025_6524_Fig3_ESM.png]

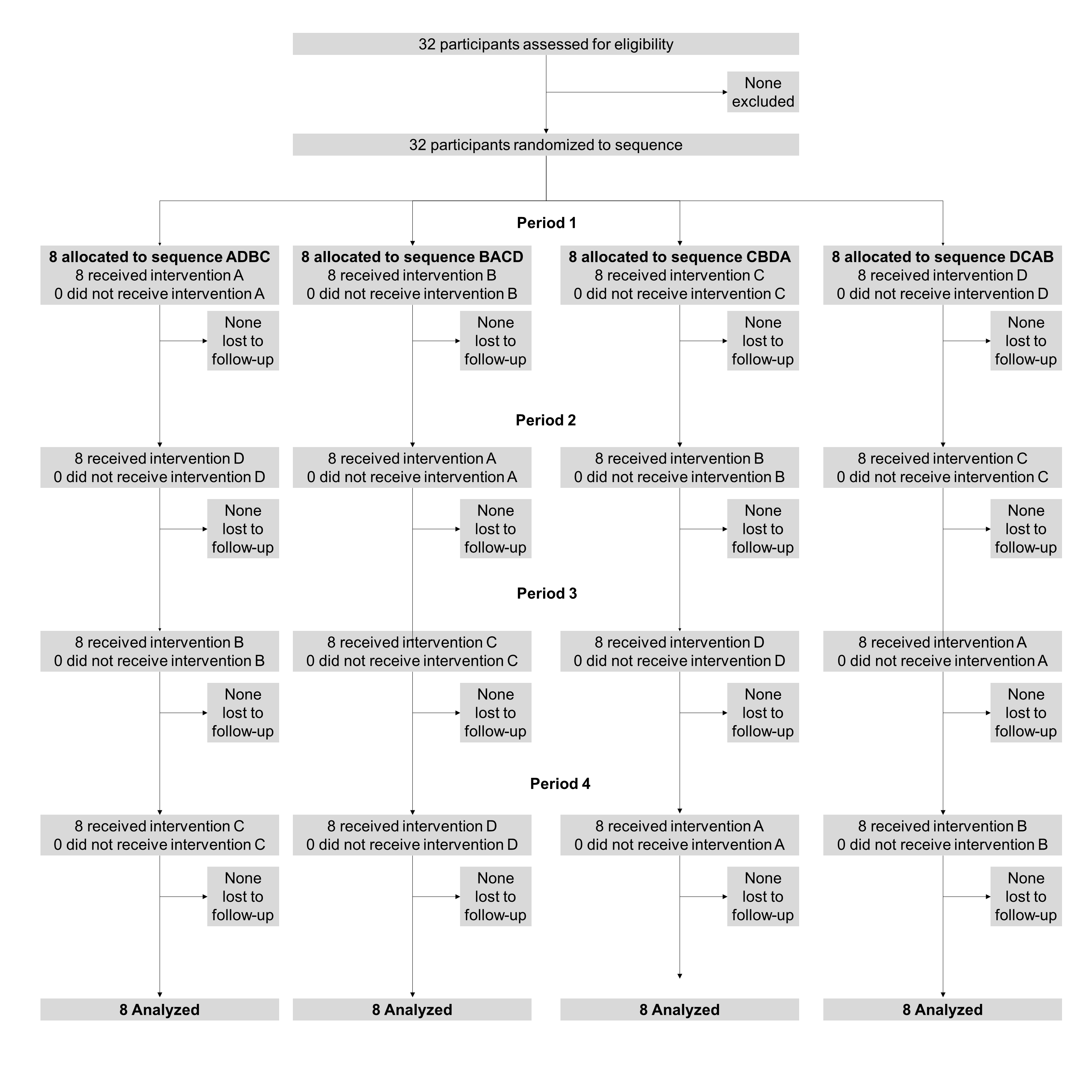

Supplement: Supplementary file 2 — High resolution image (TIF 995 KB) [file 431_2025_6524_MOESM1_ESM.tif]
